# Perception of body donation among the Phase-1 medical students, a questionnaire-based study

**DOI:** 10.12688/f1000research.175620.1

**Published:** 2026-01-29

**Authors:** B. V Murlimanju, M. Praveen Shenoy, Sheetal D Ullal, Rajanigandha Vadgaonkar

**Affiliations:** 1 DEPARTMENT OF ANATOMY, Kasturba Medical College Mangalore, MANIPAL, India; 2 DEPARTMENT OF PHARMACOLOGY, Kasturba Medical College Mangalore, Manipal, India

**Keywords:** Altruism, Cadaver, Human Body, Medical Education, Sustainable Development Goals

## Abstract

**Background:**

This study aimed to examine the knowledge, attitude, and perception of body donation among Phase-1 medical students at our institution. The objectives were to assist teachers in providing insight into the perception of this among students.

**Methods:**

This was a cross-sectional, institution-based, time-bound study comprising 29 validated questions regarding comprehensive aspects of body donation. There were 396 medical students in Phase-1 from admissions for two consecutive years.

**Results:**

Most students (94.4%) were aware of the source of cadavers in the dissection hall, which were either donated or unclaimed bodies from the hospital. However, 38.9% of them were unaware of the guidelines for body donation, and 79% were ignorant of the documents required to pledge. However, 84.3% were aware that it was mandatory to sign a pledge form. The time frame to procure the dead body to the department of anatomy of the institution was not known to 79% of the participants, and 93.2% of the participants opined that corpses from other neighboring states could be accepted. Opinions regarding the acceptance of donated bodies afflicted with long COVID were supported by 218 (55.1%), not supported by 47 (11.9%), and neutral by 131 (33.1%) participants in this study.

**Conclusion:**

Specific knowledge gaps were encountered, including the timeframe, logistics, and legal issues in procuring the body. Since medical students play an important role in this societal motivation, it is suggested that a scientific session be planned in the curriculum of Phase-1 regarding the protocol of body donation. Apart from providing insights, this study also accomplishes the United Nations’ sustainable development goal-4 in offering quality medical education. This study, apart from providing insights, also accomplishes the United Nation’s sustainable development goal-4 in offering quality medical education.

## Introduction

Presently in India, there is dearth of cadavers and there exists a need for them, to meet with the increase in the number of medical and allied health professional institutes in recent times. Significant challenges are posed due to the limited number of cadavers. The scarcity of cadavers, because of logistical difficulty in procuring, has significantly paved the way to the gradual shift in the mode of teaching to various alternatives. However, advanced multimedia technologies are unfeasible in alleviating such shortcomings due to their higher cost and maintenance in many developing countries. It is known that the practice of teaching and scientific research by cadaveric dissection has been critical, indispensable and is deeply embedded in the tradition of medical education of all disciplines. The manual cadaveric dissection aids medical students to understand the topographic localization of structures and develop a spatial and tactile appreciation of the human body, which cannot be achieved through technologically mediated learning. This forms the key cornerstone in imparting the anatomy education, although computer-based multimedia programs like e-learning modules, three dimensional animations, dissection videos, synthetic cadavers and virtual reality have gained popularity.

Apart from undergraduate medical education and scientific research, cadavers are also used to practice surgical skills and develop new techniques. Donated bodies are used for various cadaveric workshops for postgraduates, paramedics, superspecialists, and skill training. Surgically oriented departments such as obstetrics and gynecology, orthopedics, otorhinolaryngology, general surgery, oral-maxillofacial surgery, and many more require cadavers, as they conduct live workshops to improve surgery-based skills. In most medical colleges, there are well-organized anatomy museums that showcase dissected and well-mounted human bodies that educate common people. Dissection oriented competitions are often conducted among medical students to hone their surgical skills and motivate passion in this field. Anatomical fraternity across the globe approves cadaveric dissection as a supreme teaching tool in medical education and health sciences.

Voluntary donated bodies and unclaimed bodies received from hospitals were the main sources of cadavers. The first voluntary body donation documented in India was in 1956 from the Late Shri Pandurang Sridhar Apte, which was donated to B. J. Medical College, Pune, Maharashtra, India. Voluntary body donations play a pivotal role in curbing the shortcomings of cadaver procurement. However, the attitude and perception of body donation vary widely among individuals and communities according to their demographic profile and cultural acceptance. It is suggested that keeping an open mind and a positive outlook are essential when it comes to making this decision. This mindset helps individuals be pre-informed and make compassionate decisions that would contribute to a broader cultural shift in acceptance and altruism in the medical field, which is a highly valued acknowledgement and an ultimate contribution to society. This will eventually contribute to the genesis of newer and better regimes for diagnosis and patient management by students. This fulfills the donor’s ultimate altruistic move, which is considered as a ‘noble deed for a noble cause.’

Acknowledging body donation as a gracious act towards medical science is still a matter of concern, as many are ignorant of its potential significance. Body donation awareness among the general population in India is relatively low. For instance, only 32.1% of the population in Maharashtra were aware of body donation programs. ^1^ This unawareness quotient cannot be denied because most medical colleges face cadaver shortages, which are further worsened by the increase in the number of medical and paramedical courses. Healthcare professionals play a crucial role in promoting body donation; however, their knowledge and attitude need to be increased. There are specific knowledge gaps in the guidelines of body donation among students, which range from ethical and legal frameworks to post-donation processes, communication, and psychological concerns. For instance, in Italy, only 14.9% of health sciences students are aware of the relevant legal aspects of body donation. ^2^

To overcome cadaver shortages and maintain the quality of medical education, research, and surgical training, the appositeness and significance of body donation should be emphasized in society. One of the prime knowledge gaps identified in the body donation protocol is insufficient educational exposure, and there is a need for a more structured platform to deal with ethical procedural and emotional aspects. This can be achieved by conducting body donation camps, public meetings, active involvement of youth and non-government organizations, and electronic media such as radio, television, social media, and newspapers. In literature, studies on knowledge, attitude, and perception of body donation expose several significant gaps, which should be addressed to improve body donation numbers and educational outcomes. This is because the deficient body donor numbers indirectly compromise the quality of medical training, as they block the opportunity for student exposure to perform the dissection.

Donated bodies are used in biomedical research to enable health scholars to learn new techniques throughout their careers. As donated bodies provide realistic training in surgical anatomy fields and enhance medical education, they form an important component in accomplishing sustainable development goal-4 (SDG-4). This goal focuses on quality education and ensures a lifelong learning opportunity, which promotes research and innovation. Overall, this goal instills respect, empathy, and ethical responsibility in medical scholars aligning health literacy with the values of human-centered education.

Awareness of body donation among undergraduate students navigates through social responsibility to promote altruism and has a positive impact on the community by increasing donor registrations and sustaining the critical need of the hour. ^3^ Generating public awareness campaigns for body donation by undergraduate fraternity involves a balanced mix of education, engagement, and empathy. Medical students should be knowledgeable about body donation because it significantly enhances their anatomical education, fosters ethical training and interdisciplinary learning, and encourages collaboration towards professional development. Additionally, their advocacy towards this noble gesture and the regular conduct of awareness campaigns can bridge the gap in cadaver shortage and dispel myths regarding cultural taboos and religious cults. ^3^ However, the available literature is scant and does not reveal enough information regarding the knowledge and perception of body donation among medical students from a sample Indian population. Based on this rationale, the present study was undertaken to assess the knowledge, attitude, and perception of body donation among Phase 1 medical students at our institution, and to provide educators with insights into students’ perspectives on body donation, thereby aiding in the development of more empathetic and informed approaches.

## Methods

### Participants

This was a cross-sectional institution-based time-bound study, which included 396 students in Phase 1, Bachelor of Medicine, and Bachelor of Surgery from admissions of two consecutive years. Students of senior batches allied health professional courses, and those under 18 years of age were excluded from the present study. This study is from a well-established medical institution, the ‘Institute of Eminence,’ which is the pioneer in India’s public-private partnership model for healthcare and medical education.

### Ethical statement

The authors of this manuscript state that ethical approval was obtained prior to the commencement of the study. This study was approved by the Institutional Ethics Committee, Kasturba Medical College, Mangalore, India, Reg. No. ECR/541/IND/KA/2014/RR-20 (Approval number: IEC KMC MLR 05/2024/283, Dated 23/05/2024). The protocol of this study was archived in the https://dx.doi.org/10.17504/protocols.io.n92ld6kwng5b/v1.

### Procedure

A total of 29 validated questions ( Table 1) on the concepts of knowledge, attitude, and perception of body donation were administered electronically via Google Forms, and the responses were collected. The questionnaire was divided into six separate sections that included awareness, cadaveric dissection, guidelines, logistics, associated comorbidities, and incentivization ( Table 1). The electronic form began with a brief description of the criteria for participation with respect to the goals of this study. The choice to participate in the study was optional and was not compulsory. The liberty of skipping any section of the questionnaire and freedom to withdraw as participants at any point was considered. Prior to the study, each participant agreed to be a part of this questionnaire-based study and handed written informed consent. They also affirmed to publish the information data, provided the anonymity of their identity was maintained. Participants who were below eighteen years were considered as minors and thereby were excluded from this study. The name, gender, religion, and other personal student-related details were excluded and anonymity was maintained.

**Table 1.** Questions administered through electronic form in this study.

No.	Question/Opinion	Answer/Option
**Section A – Awareness of concept of body donation**		
1	Are you aware of body donation?	yes/no
2	If yes, knowledge about the body donation was obtained from	medical school/family/relatives/friends/celebrities/newspaper/television/journals/social media/others
3	If the answer to the above question was others, please specify it	yes/no
4	Can a person of age less than 18 years have the right to donate the body?	
5	Are you aware of the concept of whole-body donation?	
6	Willingness to donate to your body later in life?	
7	Will you educate your family members or relatives for body donation?	
8	Do you have any members in the family who have pledged to the body for donation?	
9	Are you interested in conducting body donation awareness programs?	
** Section B – Body donation and cadaveric dissection**		
10	What is the source of cadavers in anatomy dissection hall?	donated bodies/unclaimed bodies/both/not aware
11	Are u aware of cadaver shortage in medical colleges?	yes/no
** Section C – Guidelines for the body donation**		
12	Are you aware of guidelines of body donation?	yes/no
13	Are you aware of pledging self-body for donation?	
14	Signing a pledge form for voluntary body donation is	mandatory/non mandatory
15	Are you aware of the requirement of documents needed for pledging?	yes/no
16	If yes, what documents are required?	Aadhar card/birth certificate/voters ID/driving license/passport/employer ID/vaccination card/details of comorbidities
17	The time frame to procure the donated body is	6 hours/12 hours/24 hours/48 hours
18	The time frame to procure the unclaimed body is	
17	The time frame to procure the donated body is	6 hours/12 hours/24 hours/48 hours
18	The time frame to procure the unclaimed body is	
19	Can Autopsied bodies be accepted?	yes/no
** Section D – Logistics of body donation**		
20	Do you know about authorities/agencies who could be approached for the body donation?	yes/no
21	Can a body from another state accepted?	
22	Transport expenses of the body from another district must be borne by the institute	yes/no/depends on the institutional guidelines
** Section E – Incentives for body donation**		
23	Is it a good idea to incentivize the donor?	yes/ no/neutral
24	Is it a good idea to issue a ‘donor card’?	strongly agree/agree/neutral/disagree/strongly agree
** Section F – Donor with comorbidities**		
25	Can people living with HIV donate body?	yes/no
26	Can Individuals infected with hepatitis-B donate their body?	
27	Opinion regarding acceptance of a body of a COVID-19 death?	
28	Can people affected by chronic skin disease donate their body?	
29	Can a long-standing COVID-19 person donate their body?	yes/ no/neutral

## Results

**Figure 1.**
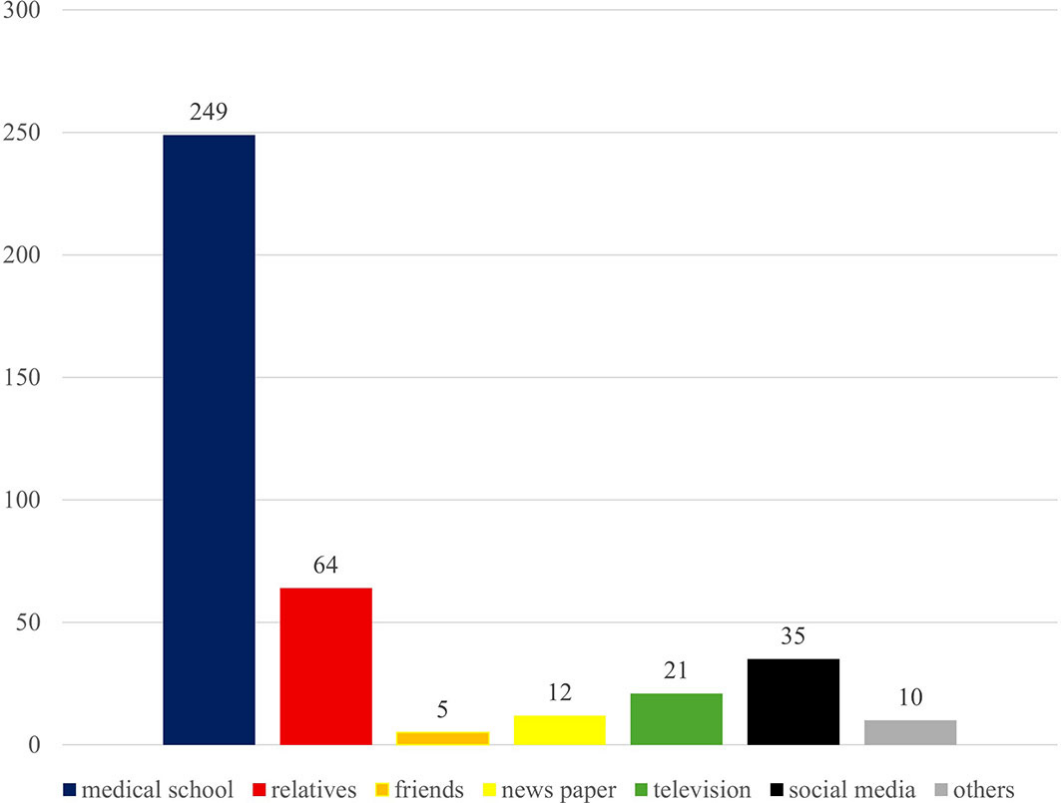
Pattern of source of information received about body donation among the participants (n = 396).

It was observed that 391 participants (98.7%) were aware of body donation. Figure 1 shows the pattern of the source of information received regarding body donation. Medical schools (63.7%) had the maximum awareness of this concept, with minor contributions from family, friends, and multimedia. In our study participants, 276 (69.7%) were aware that a minor who was less than 18 years of age had no right for body donation, and 152 (88.4%) were aware of whole-body donation. Voluntary willingness to donate to their own body in old age was shown by 220 (55.6%) students, and 145 (84.3%) showed interest in motivating their family members and relatives to donate. Forty participants (10.1%) already had a family member who pledged for body donation. One hundred and five participants (65%) expressed their willingness to conduct body donation awareness programs.

According to 87 (21.9%) students, the source of cadavers in the anatomy department was donated bodies, and the majority (287, 72.5%) were aware that they may be from either donated or unclaimed bodies from the hospitals. Twenty-two (5.6%) patients in the study group were unaware of the source of cadavers in the dissection hall. A critical concern regarding the shortage of cadavers in medical college teaching was reported by 320 students (80.8%). The opinions of the participants regarding the guidelines for body donation are tabulated in Table 2.

**Table 2.** Opinion of participants regarding the guidelines for body donation (n = 396).

Question	Opinion	Number	Percentage
Are you aware of guidelines of body donation?	Yes	242	61.10%
	No	154	38.90%
Are you aware of pledging self-body for donation?	Yes	279	70.50%
	No	117	29.50%
Signing a pledge form for voluntary body donation is	mandatory	334	84.30%
	Not mandatory	62	15.70%
Are you aware of the requirement of documents needed for pledging?	Yes	83	21%
	No	313	79%
If yes, what documents are required?			
Aadhar card		363	91.70%
Birth certificate		265	66.90%
Voter ID		69	17.40%
Driving license		65	16.40%
Passport		65	16.40%
Employer ID		37	9.30%
Vaccination card		209	52.80%
Details of comorbidities		241	60.90%
Are you aware of the time limitations in procuring a dead body?	Yes	83	21.00%
	No	313	79%
The time frame to procure the donated body is	6 hours	33	8.30%
	12 hours	12	3%
	24 hours	140	35.40%
	48 hours	211	53.30%
The time frame to procure the unclaimed body is	6 hours	63	16%
	12 hours	125	31.60%
	24 hours	70	17.70%
	48 hours	138	34.70%
Can autopsied bodies be accepted?	Yes	254	64.10%
	No	142	35.90%

**Figure 2.**
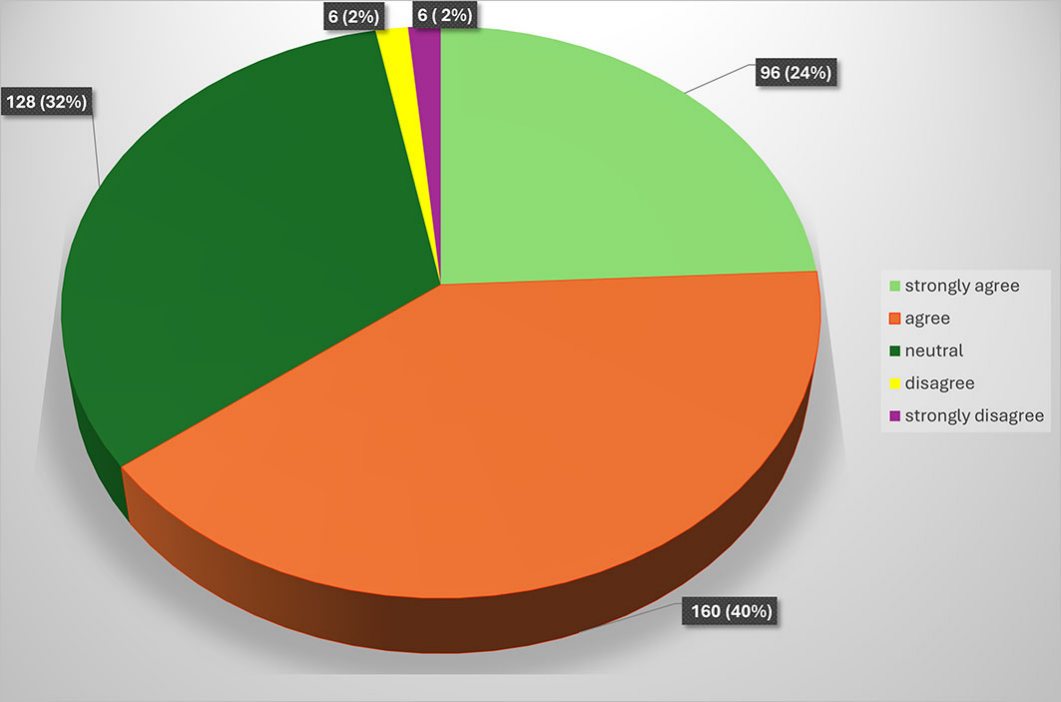
Frequency of opinions regarding issuing a donor card among the participants of this study (n = 396).

Only ninety-nine of the study participants (25%) had knowledge of organizations, who could be approached and concerned with dos and don’ts of body donations. The majority of participants (369, 93.2%) opined that the corpse could be accepted from other neighboring states. Two hundred and twenty-three (56.3%) expressed that the institute must take the responsibility of expenditure of transporting the cadaver from the neighboring state, and 13 (3.3%) participants denied this. However, 160 (40.4%) patients opted that this depended on the guidelines of the respective institutions. The concept of body donation incentivization was supported by 122 (30.8%) participants, and 111 (28%) did not agree. However, 163 (41.2%) participants were neutral about the donor’s rewards. The frequency of opinions regarding issuing a donor card is schematically represented in Figure 2.

Among our participants, 182 (46%), 183 (46.2%), 231 (58.3%), and 198 (50%) opined that people living with HIV, hepatitis B, chronic skin diseases, and death due to COVID-19 could donate their bodies, respectively. Opinions regarding the acceptance of donated bodies afflicted with long COVID were supported by 218 (55.1%), not supported by 47 (11.9%), and neutral by 131 (33.1%) participants in this study.

## Discussion

Body donation or bequeathal is the informed and voluntary act of giving away one’s own body after death for medical education and research without any expectations or rewards. It is a vital source of cadavers for medical training and is considered an altruistic act of significant moral value. As medical education has evolved, standardized, and revised, cadaveric dissection has become the core part of laying foundations in medical curricula. A direct hands-on experience with the human body holds an upper hand for medical and surgical training. Far from becoming obsolete due to legislative insufficiency and financial constraints, dissection has retained its significance alongside scientific advancements, reinforcing its crucial role in medical training. The key to training a medical scholar is always classical cadaveric dissection, which highlights the topographical and spatial understanding of the region and plays a pivotal role. It is of utmost importance to model a competent professional with an innate foundation of anatomy and medical-related perceptions. Lack of adequate knowledge of anatomy and its applied concepts contributes to iatrogenic causes of human morbidity and mortality. The anatomists at the Royal College of Surgeons highlighted the need for basic anatomical knowledge that could prevent serious complications in clinical medicine. ^4^ It boosts the confidence and competence of a medical professional towards preventing potential risks to patient safety. Lapses made during surgical procedures, faulty diagnoses, or even incorrect treatment regimens led to gaps in anatomical knowledge, emphasizing the critical need for robust and comprehensive anatomical teaching in medical education, which is achieved only by direct exposure to human cadavers. For medical and paramedical scholars, cadaveric dissection remains the best method for teaching the human body, as it works on the sensory, graphical, kinesthetic, and psychological aspects. The medical curriculum under the new competency-based medical education for Phase-1 medical students has developed a special module that emphasizes the importance of cadavers and related issues.

Dissection offers a direct hands-on understanding of the human form and provides three-dimensional appreciation, but the demand for cadavers often exceeds the supply. With the increasing use of cadavers in medical science and research, there has been a significant increase in the number of medical colleges. Body donations are essential for dissection-based anatomy teaching, but there are various factors that could impact the willingness of individuals to donate their bodies, which in turn affects the availability of cadavers for medical education. Several studies have explored the factors that influence people’s willingness to donate their bodies. These factors include ancestry, ethnicity, demographic characteristics, awareness, age, gender, education level, occupation, income, religious beliefs, marital status, and number of dependents. A survey conducted in China, on the usage of cadavers in teaching anatomy, found that the cadaver resources of most medical schools are associated with geographic location, academic ranking, and local support for body donation policies. ^5^ Despite body donation being the preferred and major source for cadavers, the percentage of the general population willing to donate the body is meagre. Boulware et al. ^6^ found that in African Americans, older individuals with lower education, lack of insurance, unemployment, coexisting medical conditions, and religious concerns were the reasons for scant donations. Cultural beliefs and ethical concerns significantly impact the willingness to donate; for example, concerns about post-mortem bodily integrity and religious beliefs are major barriers. In Rwanda, despite the good knowledge scores, 66.7% of students were unwilling to donate their bodies, highlighting the influence of cultural factors. ^7^ It was reported that there is a notable gap between the awareness of body donation and actual registration of donors. For instance, in a study among Indian medical students and professionals, 46.3% had a positive attitude towards body donation, but none had filled out a pledge form. ^8^ Similarly, in Poland, only 24% of students were willing to register as donors despite being aware of body donation and its benefits. ^9^

Educating medical scholars about body donation is an important practice, as it not only represents a potential donor population, but also plays a key role in promoting public awareness as future healthcare educators. In a pilot Italian study by Ciliberti et al. ^10^ it was observed that despite approving cadaver dissection as an important part of their training, the medical students were still unaware of the in-depth knowledge of this noble practice, suggesting a significant cognitive gap.

Due to the sudden surge in the requirement of cadavers and to legalize the process of procurement, various laws were passed by governments concerning obtaining bodies for anatomical dissections, which were later disseminated through various anatomical acts. Anatomical dissection of cadavers requires clearly laid rules that direct and limit their legal boundaries, thereby framing them from a bioethical perspective. To begin, the British government passed ‘the Murder Act of 1752,’ which is one of the earliest references regarding the law concerning cadaveric procurement that permitted the public dissections of executed prisoners. It also became quite effective in reducing the dreadful crimes of body snatching and provided a baseline to build on ethical issues regarding the same. In contrast, there were loopholes leading to its failure, such as who should be designated to conduct dissections and the need for a licensed governing body. ^11^

In India, to fulfill the cadaveric demand of a medical institute, the Anatomical Act was formulated and implemented in various states, which underwent multiple revisions with different versions and published in the respective state government gazette. The ‘Bombay Anatomy Act-1949’ ^12^ which was formatted from Coroners Act of 1871 and is now presently referred as ‘The Maharashtra Anatomy Act,’ was the first legal anatomical act in India. This Act legally permitted the supply of unclaimed bodies to hospitals and medical colleges. This act highlights the ethical concerns of both unclaimed and voluntary donated bodies and is adopted uniformly in all states of India. This act authorizes physicians, surgeons, anatomists, and medical students to implement dissections on voluntarily donated bodies for scientific studies. As per this Bombay Anatomy Act, unclaimed bodies can be collected by the Department of Anatomy if the death occurs in a hospital of the same state or in a public place within the periphery of that medical college, provided within 48 hours of death and no claimant has been declared by the police. Most of the participants in the present study (93.2%) opted that their bodies could be accepted from other neighboring states. However, the Anatomy Act in India is state-specific, and the acceptance of bodies from another state might rely on the guidelines of the receiving state. ^11^ There is a requirement for a unified Anatomy Act in India to centralize the process of body donation at a national level. Their suggestions can prevent disparities across states in India and ensure uniformity in the procurement of bodies from other states.

The general population should be made aware of the legal issues of voluntary body donation that could deter a willful donor. Specific programs are designed to allow medical professionals to promote better public recognition and awareness of whole-body donation. However, Bolcato et al. ^2^ noted that 14.9% of respondents had poor knowledge of this new Italian law. In the present study, 38.9% of participants were not aware of the guidelines of body donation, which is a higher frequency than the findings of Bolcato et al. ^2^ In the present study, 70.5% of participants were aware of pledging the self-body for donation and 84.3% stated that signing a pledge form for voluntary body donation is mandatory. Brenner et al. ^13^ updated in his work that only 18 European countries are governed by national regulations, with notable variations in the legal and ethical frameworks governing the donation. The criticality of informed consent, body donation protocol, eligibility criteria, and handling of the body to the final disposition of the remains are the most fundamental requirements. In France, the centrality of donors’ consent in written form is often referred to as a gift document. The gift document includes all personal details, eligibility criteria, logistics, legal and financial aspects, and the final disposition of the remains with their signatures. Despite significant variation in the guidelines for body donation across different countries and continents, there are some best practices that can still be identified. Sink et al. ^14^ revealed that there are significant differences in thoughts about body donation based on religious differences and the background of anatomy education programmes. A lower frequency of interest in body donation was observed among Catholic students as compared to agnostic and atheist students. Despite supporting body donation, the registration rates among Slovenian students were low, which calls for raising the bar for ethical awareness and motivating body donation. In a study conducted by chiropractic students at Macquarie University, religion was reported to play an important role towards the opinion of body donation. In the study by Sink et al. ^14^ 33.1% of students were interested in donating their own bodies later in life. It was reported that students with a prior anatomical background were inclined towards organ donation rather than body donation due to concerns regarding handling of pre and postdissection. ^14^ In the present study, the ethnicity and religion of the students were not taken into consideration. As these anatomical acts are enacted in different states, nations, and countries, unclaimed bodies can be legally procured by medical institutes and used for dissection and learning. It is now mandatory for any medical teaching institute to acquire legal approval for anatomical acts by a government authority to permit the dissection of a dead body for teaching or research intent; otherwise, it would be considered an illegal offence. Currently, body donation programs are organized in the United States, and they are the source of cadavers in American medical schools. Like the United States, Asian countries have also faced a shortage of cadavers.

Saha et al. ^8^ conducted a study to evaluate the breach between awareness and actual participation in donation and noted that 18% of the participants were against body donation. There are several factors responsible for the denial of donating, and the most important factor is lack of awareness. In a study conducted by Rokade and Gaikwad, ^1^ it was observed that only 32.1% of the general population was aware of body donation compared to 95.8% of healthcare professionals. Medical school students gain knowledge of body donation through the amalgamation of educational programs, institutional practices, and public awareness initiatives. In the present study, 98.7% of students were aware of the concept of body donation. This frequency matches the results of a study by Behera et al. ^3^ who reported that 96.7% and 93.2% of medical and other health professional course students, respectively, were aware of this concept. The frequency of knowledge of body donation awareness in this study was like the frequency found among Nepalese students. It was reported that 93.5% of medical and nursing Nepalese students were aware that a person can donate the body for educational purposes. ^15^ In many countries promotion of body donation is being made through community awareness programs, commemoration services for the donor families and incorporating this concept into medical curriculum through educational initiatives. ^16^ The study by Vadgaonkar et al. ^17^ reported that Phase-1 medical students were aware of the ethical guidelines of cadaveric dissection, and they acknowledged that cadaver is their first teacher. This suggests that cadaveric dissection emphasizes the importance of body donation, and medical schools have become their source of information. Information on body donation can be published on the website of the institution’s anatomy department.

Relatives and family were the sources of information on body donation for 64 (16.4%) participants in this study. It is important to note that few of the students in this study were children of doctors, and they were their source of knowledge. Social media was the source of information on body donation for 35 participants (8.9%). The importance of social media is that it quickly disseminates information and reaches the maximum population. Television, newspapers, and friends were the sources of information for 5.4%, 3.1%, and 1.3% of the participants, respectively. Ten participants in this study (2.5%) received information about body donation from other sources such as Google and other online resources. In the present study, 69.7% of the participants were aware that a minor could not give consent for body donation. Body donation involves informed consenting in several nations; for example, The Human Tissue Act 2004 in the United Kingdom strictly insists on informed consent for organ donation. This suggests that there is an age restriction, and minors cannot give consent for donation. Anatomy acts in India and insists on informed consent, and the requirement of witnesses from relatives is mandatory for body donation. However, there are a few exceptions, such as in the Kerala and Delhi states of India.

Few studies suggest that there is a scarcity of comprehensive knowledge about the guidelines of body donation ^18^ and that awareness of the same varies among medical professionals. In the present study, 88.4% of the participants were aware of whole-body donation, and 55.6% expressed the voluntary willingness to donate to their own body in old age. This incidence is higher in comparison to the previous study by Behera et al. ^3^ where this was observed in 34.6% and 56.3% of medical and paramedical students, respectively. Ballala et al. ^18^ reported that only 22% of the medical professionals were willing to donate their bodies. However, 68% were interested in insisting that the public make the donation. It is suggested that the anatomy department faculty and dissection technicians should undergo programs on soft skills in communication with potential donors in the process of body donation. ^19^ One hundred and five (65%) of our participants expressed concerns in conducting the body donation awareness programs. Medical students with a positive attitude towards body donation are more likely to influence and promote it within their families and peers. It is suggested that faculty and medical students work together to raise awareness quotients of body donation programs. The best example of an approach by the students to conduct awareness is an Italian study, where the sets created a graphic medicine project to increase awareness of body donation. ^20^

Sink et al. ^14^ reported that 26.3% of students would recommend and advocate body donation to a family member. In our study, this frequency was higher (84.3%), as 145 participants showed interest in motivating their family members and relatives to donate. Forty participants in this study (10.1%) had a family member who had pledged for body donation. Family members play a significant role in this process.

It takes almost two–three years before the dissection is completed, and appropriate disposal of the cadaveric remains. ^21^ Most of the unwilling participants felt that the donated bodies were misused or abused. Psychological anxiety topped with family pressure was also attributed to this unwillingness. Cahill et al. ^22^ conducted a survey among medical students to measure their attitudes towards dissection as an aid to education and whole-body donation. It was hypothesized that medical students, given their exposure to the importance of cadaveric dissection, would be more supportive of body donation than the general population. However, they reported that despite students being aware of the benefits of cadaveric dissection, there was a dip in the percentage of the idea of donating their own body, and they were also unlikely to motivate their families for the same. According to 72.5% of our students, the source of cadavers in the department of anatomy was donated or unclaimed bodies from hospitals. This suggests that they are aware of the source of the cadaver, which they dissect to gain knowledge of human anatomy.

The frequency of cadaver source varies across nations. Unclaimed bodies are the main source of cadavers in countries like Turkey (84.8%) and Nigeria (more than 90%); however, North American countries procure cadavers by body donation (80%) ^23^ of the critical concern regarding the shortage of cadavers in medical college teaching was reported by 80.8% of the students of our study. This frequency is higher than that of 45% of participants by Behera et al. ^3^ enhancing public awareness and fostering positive attitudes among the students regarding body donation can effectively mitigate the shortage of donated bodies.

It is essential to recognize the barriers and impart proper guidance that prevents individuals from filling out the pledge forms despite knowing the importance of donation. A questionnaire-based study, conducted to evaluate the attitudes of Turkish anatomists towards the cause of insufficient cadavers, was attributed to the decreased availability of unclaimed bodies and body donors. ^24^ It was proposed that there should be an increase in the intake of unclaimed bodies and body donation awareness campaigns to overcome this insufficiency. Since the unclaimed dead bodies received are not sufficient to meet the increased demands, it warrants the body donation movement to be geared up. The religious support and ethnicity of the different sects are critical, as there are innumerable people active in practicing their respective religions. Therefore, it is crucial to understand the reasons behind the unwillingness towards body donation based on religious or cultural beliefs.

Medical colleges have their own procedures and policies for body donation, which include inquiry, registration with the department of anatomy, filling the consent form, documentation, and fulfilling requirements such as death certificates and transportation arrangements. Specific guidelines vary between the institutions of different states and nations. There are no specific guidelines for body donation from the National Medical Commission of India. Hence, it is important to consult the chosen medical institution to obtain detailed information regarding body donation. There is no upper age limit, although the legal minimum age for body donation is 18 and above in India. A detailed body donation protocol should be explained to the donor or family members, and they should be informed that the body should be used for dissection, teaching, and anatomical research, and that the remains of the cadaver will be scientifically disposed of later. The body donation procedure begins with registration with the department of anatomy of a medical institution or a recognized body donation organization, with signed written informed consent from the donor or their legal heirs or near relatives, and the witnesses. Consent forms are available at medical institutions, non-governmental organizations (NGO’s), or can be downloaded from the official website. If death occurs due to natural causes or in a hospital, and if it is not a medicolegal case, the body can be handed over to the department of anatomy. However, when the death occurs at home or anywhere else, other than a hospital, intimation to the police station can be given. The police can be informed of the medical institution to which the body donation is pledged for official records and future references. A ‘no objection certificate’ should be issued by the police station in case of a medicolegal situation. Established NGO’s working in the field of body donation can act as facilitators between the donor family and the medical college. Transparency and clear communication of information need to be maintained between the institution and potential donors and their nearby relatives right from the receipt of an initial inquiry until the final disposal of the remains. This includes the procedure of obtaining information at the time of death, transporting the body to the medical institution, and the documents required to be submitted. Details of the documents required in the process must be iterated to the donor and family personnel, such as the death certificate, which is issued by a registered medical practitioner stating the cause of death.

An interesting finding was noted in the present study, in which only 21% of the participants were aware of the details of the documents required for pledging. We suggest that a timely sensitization regarding the documentation involved in the process of body donation should be offered to young medical scholars, which would motivate them to create awareness in their families and the public. It should be noted that the mandatory requirements of body consent include a duly self-filled and signed form appended by the witnesses’ signatures along with the death certificate. Adhar card and other relevant identity proofs of the deceased and consenting family members to be deposited. In a few developed nations like France, the death certificate should also rule out any medico-legal issues and potential communicable diseases of the donor. ^25^ The participants of this study opined that, birth certificate (66.9%), voter identity cards (17.4%), driver’s licenses (16.4%), passports (16.4%), and employer identity cards (9.3%). However, these methods have not been universally mandated. The focus of the requirement of the driving license document is on organ donation rather than body donation. Vaccination cards (52.8%) and details of comorbidities (60.9%) were also suggested by our participants. These may be relevant in this post covid era as few institutions may necessitate COVID vaccination certificates.

The transfer and preservation of the body donated to medical colleges is a crucial part of the body donation ecosystem, which is often underfunded or overlooked. Logistics, such as timely transportation of the body from the place of death, resources, and infrastructure of the medical institution in handling the body after receiving are the often-underrated barriers to body donation. In this context, every medical institution should ensure the maintenance of the best infrastructure required for body preservation, and train anatomy technicians for embalming. Due to these factors, some institutions may be unable to accept the body, even if people are willing to donate. The distance of the medical college from the place of death, maintenance of the timeframe, lack of trained staff, and specialized mortuary vehicles are some of the logistic challenges faced. On many occasions, the donor’s family does not have access to such services, and a short time frame can be logistically confusing. They can also be financially unaffordable and emotionally difficult during grief. In this context, strengthening the logistical infrastructure is vital and making body donation accessible, without which the generous wish of the donor may go unfulfilled. Medical colleges can have an effective transport system by collaborating with private ambulance services, NGOs that work with health logistics, and funeral transport companies. The body must be transported to the designated medical institution as soon as possible, preferably during the working hours. In this study, 8.3% and 3% of the participants opined that the body could be transported to the department of anatomy within 6 h and 12 h of death of the donor, respectively. However, 35.4% opined that the time frame was 24 hours, and 53.3% said that it was up to 48 hours. The same participants opined that the time frame was 6 hours (16%), 12 hours (31.6%), 24 hours (17.7%), and 48 hours (34.7%) for receiving an unclaimed body from the police. According to the literature, the preferred timeframe is within six hours and in case of delay, the body may need to be kept in the cold storage or morgue of a nearby hospital. The post-mortem changes start appearing within two hours and post-mortem fixation occurs by 9 to18 hours, which suggests that the body should be transported to the department of anatomy as early as possible for embalming and preventing decomposition. ^26^ On some occasions, partial embalming is arranged by the relatives themselves to facilitate the last rights or tributes, arrival of foreign relatives to pay the last respects, or for some other religious purpose. It has been described that the time frame is flexible, but it is as per the guidelines of organ and tissue donation. Among the students in the present study, 64.1% opined that autopsied bodies could be accepted for dissection by the Department of Anatomy. However, the remaining 35.9% were correct, as the autopsied bodies could not be accepted universally. However, this depends on institutional policies and ethical guidelines.

Only 25% of the participants in this study had knowledge of organizations who could be approached and concerned with dos and don’ts of body donation. In India, the process of body donation is governed by the Anatomy Act, which supervises the supply of bodies to medical and other health professional colleges. Body donations in North America, Australia, and New Zealand are governed by university-based programs, which offer memorial services to donors and their families. These programs oversee the entire process, starting from body donor registration until the procuration of the body. In the United States, a relatively recent development to enhance body donation is the creation of certain organizations. These are ‘for-profit willed body companies,’ also known as body brokers. ^12^ These organizations cover all the needs of the donor family from advertisements, cremation charges, and other fees incurred, followed by distributing and charging the bodies or body parts nationally and internationally, which generate substantial profits. Champney et al. ^12^ have reviewed the evolution and legal and economic aspects of willed body programs and provided an ethical framework for their use. In Australia, there are also few body donation programs that are considered central mortuaries, and they oversee supplying bodies. ^16^

The transport expenditure of the body should be borne by the institution to respect the dignity of the donor. ^21^ In this study, 56.3% of students expressed that institute should take the responsibility of expenditure of transporting the cadaver from the neighboring state and 40.4% opted that this depends on the guidelines of the respective institution. However, according to many institutions, the possible costs incurred by the donor’s family can be compensated by the medical college by accepting the settlement if the death occurs within a kilometer’s radius from the institution and if the body is procured during working hours. Presently, in India, most medical colleges are not taking the responsibility of paying the transportation charges of procuring bodies from neighboring states and districts due to litigation and logistics issues.

Death due to natural causes, such as old age and non-communicable diseases, are accepted for donation. Unnatural deaths in which inquests have been carried out, with extensive burns, suicide, homicide, and other medicolegal cases, are not ideal for body donation. The bodies that are severely damaged in road traffic accidents, unnatural death, or an inflicted body due to infections such as active tuberculosis, hepatitis B, HIV, and COVID-19 should not be accepted by the medical institute, as they carry the potential risk of health hazards to the staff, faculty, and medical students handling them. Individuals with active skin lesions, infections, or malignancies should undergo thorough screening and risk factor analysis for organ or whole body donation, to ensure the safety and success of the procedure. Hence, it is essential to take the details of the donor or body and check whether they are eligible for donation. Among our participants, 46%, 46.2%, 58.3%, and 50% opined that people living with HIV, hepatitis B, chronic skin diseases, and death due to COVID-19 could still donate their bodies, respectively. Opinions regarding the acceptance of donated bodies afflicted with a long COVID were supported by 55.1% of the participants. However, there are opinions that these bodies cannot be accepted in India. The main concern with the body of a person living with HIV is the potential risk of transmission of the infection to the staff, who is the first point of contact. However, this risk can be decreased under stringent aseptic precautions and specific protocols. However, there are no clear rules on the practice of body donation by HIV-infected individuals and hepatitis-B in India, although this concept defers organ donation. Despite the prevalence of life-threatening viral infections such as HIV, hepatitis B, and SARS-CoV-2, there are studies being reported only for COVID-19, where the individual’s post-recovery can be qualified for whole-body and organ donation.

During the first wave of the COVID-19 pandemic, body donation programs were cancelled because of concerns about the communicability of SARS-CoV-2, which led to the decreased procurement of bodies. ^27^ The bodies were not accepted, keeping the safety of students and health care workers, as there was no screening available to rule out COVID infection in the dead person. Initially, there was no approved vaccine available, which was a concern in the institutions on the safety of use of cadavers for dissection. ^28^ However, there were studies suggestive of embalming cadavers with formaldehyde that can significantly reduce the presence of viable SARS-CoV-2 RNA and neutralize them. In this context, institutions should implement laborious safety guidelines, which include posthumous RT-PCR testing of the donor and following a thorough embalming protocol. With these safety guidelines, it is possible to utilize COVID-19 positive cadavers for anatomical dissection, teaching, and research. ^29^ However, acceptance of COVID-19 infected body is subject to institutional policies and the accessibility of safety measures. ^27^ It which suggests that universally accepted guidelines should be updated and regularly monitored to ensure the safety of COVID-19 body handlers.

In India, the ‘Anatomy Act’ regulates procurement of unclaimed bodies for medical teaching and research. Unclaimed bodies are individuals who die in public places and remain unclaimed by their families or relatives for a specified period. If there are no claimants to the body and no objection or suspicion of foul play, the delegated authorities can declare it unclaimed. Once declared unclaimed, the body can be donated to a medical college under the provisions of local anatomy acts for educational purposes. Despite legal assistance, ethical concerns exist regarding the use of unclaimed bodies in terms of consent, dignity, and transparency. Therefore, there is a required drive towards increasing voluntary body donations to reduce dependency on unclaimed bodies. To increase cadaver numbers and maintain the standard of medical education, the importance of body donation should be emphasized to the public. Structured training interventions can improve knowledge and attitudes towards donation; however, they mandate the need for continuous education to maintain knowledge levels.

Research indicates that willingness to donate one’s body is influenced by age, with younger individuals showing a greater inclination toward body donation than older individuals. Likewise, males have a more positive attitude towards body donation than females, and the educated class is more willing to donate than the uneducated class. Matheson et al. ^30^ reported that there was a difference in opinion regarding the dissection of unclaimed bodies, where 30% of students found the exercise to be ethical and 47% of them considered it unethical and expressed negative attitudes. Over the years, the concept of body donation has evolved with differences in opinions among various nations regarding the procurement of cadavers. The International Federation of Associations of Anatomists suggests that only donated bodies should be used for teaching and medical research. The available literature reveals that anatomists also depend on unclaimed bodies, importing cadavers from the United States, India, or bodies of executed persons. In many countries, body donation is unfeasible because of cultural or religious factors. Developing culturally sensitive educational programs and interventions can address specific barriers related to cultural and religious beliefs in these countries.

Regarding the incentivization of the donor, the medical college can issue a donor card or an acknowledgement letter of thanks and appreciation, which can be brought by the family member along with the body of the donor as proof of body bequeathal. In the present study, 64% of participants favored donor card issuance. The donor card also facilitates the donor’s interest and consent, fulfilling the fact that the body will be used as per their last wishes. ^25^ In few nations, donors or their family members are publicly honored in the events hosted by the NGO’s, government health organizations and other health related missionaries. They are acknowledged and rewarded with certificates of appreciation, memorial plaques, or trees planted with their names and media coverage. However, there is a difference in opinions regarding whether financial incentives can be offered to increase body donation. It is not encouraged because any financial gain or commercial dealings in body donation can create exploitation and trafficking, and it is against the altruistic nature of body donation. Only 30.8% of the participants in this study supported the concept of incentivization of body donors. This suggests that most participants were aware that incentivization has significant ethical implications. While the prohibition is for obvious reasons, incentivization encourages and allows community recognition of body donations. It is recommended that due appreciation be awarded to healthcare professionals and NGO’s, who constantly thrive in the marketing of voluntary whole-body donation and professing it as a noble act.

In many countries, voluntary donations have been guarded by ethical and legal concerns and revisited to meet the demands of teaching medicine and scientific research. As there is an increasing demand for human bodies in teaching and research, awareness and educational body donation programmes must be implemented. They should be promoted on a large scale in all groups of the general population, as improved publicity is key to increasing donations. Body donation campaigns should be appropriately designed to provide broad publicity, acknowledgement, and incentivization to the donor and their families to create and elevate awareness of this altruistic act and to change the mindset of society considering the various barriers cited for no-body donation. The altruistic decision of the donor serves a collective purpose in supporting medical advancements, surgical training, and newer innovations in biomedical research. Their legacy continues to live in every physician they train and every life that physicians go on to impact.

These regular awareness programs and outreach camps in the peripheries regarding body donation and inclusion of the same in the high school and pre-university curriculum will be of great advantage in overcoming this situation. Enhanced educational programs of this variety can implement continuous and comprehensive education, including practical training and legal knowledge about body donation. Students of the science stream should be well equipped with the awareness and benefits of whole-body donation to motivate them to inform their families and the common public to generate the willingness for body donation. Students should be taught to respect cadavers, the pricelessness of donated bodies, and the sentiments of donors behind body donation. The practice of respecting the cadaver at the beginning of the medical course and the ceremonial cadaveric oath should be emphasized mandatorily. ^17^ The message should spread about the good and respectful handling of donated bodies in the anatomy laboratory. This can have a positive impact and a sense of social responsibility, which motivates students to consider potential paybacks to medical fraternity. Effective measures to alleviate the shortage of cadavers should be meticulously planned by evoking public awareness, medical exhibitions, integrated debates, opting for a standard procedure with a clear legal framework, working on the idea of cadaver pooling, and an effective humane protocol for body donation. It is suggested that mass education is necessary to demonstrate the importance of body donation for medical education and research.

Pre-informing healthcare professionals regarding policy and regulatory guidelines is a prerequisite and of paramount importance. Addressing knowledge gaps through targeted educational interventions, culturally sensitive approaches, and enhanced public awareness campaigns can significantly improve the frequency of body donation and the effectiveness of medical education. By focusing on and addressing these breaches, we can motivate body donation and implement SDG-4 of the United Nations, which insists on quality medical education and research.

## Conclusion

The present study observed specific knowledge gaps in the guidelines for body donation, such as timeframe, logistics, and legal issues in procuring. Since medical students play an important role in this societal motivation, we suggest that a scientific session in the curriculum of Phase-1 regarding the protocol of body donation be incorporated. In this context, it is believed that this study shall sensitize the scientific community for the generous act of body donation, ‘gift that lives forever’.

## Ethical statement

The authors state that every effort was made to follow the institutional and international ethical guidelines and laws pertaining to this study. The authors of this manuscript state that ethical approval was obtained prior to the commencement of the study. This study was approved by the Institutional Ethics Committee, Kasturba Medical College, Mangalore, India, Reg. No. ECR/541/IND/KA/2014/RR-20 (Approval number: IEC KMC MLR 05/2024/283, Dated 23/05/2024). The protocol of this study was archived in the https://dx.doi.org/10.17504/protocols.io.n92ld6kwng5b/v1.

## Consent to participate

Prior to the study, each participant agreed to be a part of this questionnaire-based study and handed written informed consent. They also affirmed to publish the information data, provided the anonymity of their identity was maintained.

## Data Availability

Repository name: [Dataset on awareness of body donation guidelines]. Figshare link: https://figshare.com/articles/dataset/Dataset_on_awareness_of_body_donation_guidelines/31026607?file=60873361 with DOI: https://doi.org/10.6084/m9.figshare.31026607 ^31^ The project contains the following underlying data: [awareness of body donation guidelines] (Raw Data). Data are available under the terms of the Creative Commons Attribution 4.0 International license (CC-BY 4.0).
