# Supplementary material for: Awareness and Perception of concept of  body donation among the first year medical students: A questionnaire-based study
Source: F1000Res. 2026 Jun 1;15:133. Originally published 2026 Jan 29. [Version 4] doi: 10.12688/f1000research.175620.4 (PMC13320121; doi:10.12688/f1000research.175620.4)
Supplement: Peer review history

# Reviewer report: Reviewer response for version 4

**DOI:** 10.5256/f1000research.202037.r495569

**Published:** 2026-06-30

**Authors:** Benjamin Arko-Boham

**Affiliations:** 1 Department of Anatomy, University of Ghana Medical School, Accra, Ghana

The study is relevant and well-conceived. However, there are several factors that limit its indexing in its current form.

 1. The language requires massive editing to improve scholarship.

 2. The presentation style should be improved to enhance information flow and coherence, and to remove redundancies.

 3. Introduction: (i) The flow should be improved. The section is poorly referenced. Only three references are presented, with the first two appearing in the fourth paragraph.

 4. The Methods section: (i) It should conform to the STROBE Guidelines. (ii) The questionnaire development leaves many questions unanswered. How were the 29 items arrived at? How was content validation conducted? Was the questionnaire pre-tested? If yes, how and where? 

 5. The Results section: (i) it is largely descriptive with no inferential analyses. Frequencies should be reported with 95% confidence intervals (95% CI). (ii) Before presenting the frequencies, it will be proper to present the demographics of the participants and general participant information - how many respondents, sex, age ranges, religion, ethnicity, nationality. These are important to contextualize the findings. (iii) Some of the data in Table 2 are inconsistent. For example, only 83 of the participants responded 'YES' to knowing the documents needed for pledging. The breaking ("If YES .....") can, therefore, not exceed 83. Why the 363, 265, etc?

 6.  The Discussion section: (i) is very lengthy, convoluted, and less focused. It needs massive restructuring to focus it. (ii) The referencing should also be improved. (iii) The limitations of the study should be discussed (iv) The limitations of the study should be discussed.

Is the work clearly and accurately presented and does it cite the current literature?

Partly

If applicable, is the statistical analysis and its interpretation appropriate?

No

Are all the source data underlying the results available to ensure full reproducibility?

Partly

Is the study design appropriate and is the work technically sound?

Partly

Are the conclusions drawn adequately supported by the results?

Partly

Are sufficient details of methods and analysis provided to allow replication by others?

Partly

Reviewer Expertise:

Anatomical Education Research, Whole-Body Donation, Cancer Biology, Morphological Research

I confirm that I have read this submission and believe that I have an appropriate level of expertise to confirm that it is of an acceptable scientific standard, however I have significant reservations, as outlined above.

# Reviewer report: Reviewer response for version 3

**DOI:** 10.5256/f1000research.198077.r476028

**Published:** 2026-05-15

**Authors:** Ibe Michael Usman

**Affiliations:** 1 University of Rwanda, Huye, Rwanda

Thank you the opportunity to review the work titled “Perception of body donation among the Phase-1 medical students, a questionnaire-based study.” The topic is timely and will be interest to the scientific community. However, I have the following concerns to be addressed

 The title needs to be modified or improvements to capture what exactly was done in the current study. What does phase 1 mean? title using a more globaly acceptable terms 

**Abstract**

 The background of the study is confusing, knowledge, awareness, attitude differ from each other 

 The abstract should capture the study location or the name of the institution, when the study was done

 What does the authors mean by two consecutive years? Not clear

 The conclusion should be in line with the study finding

**Introduction**

 Grammatical issues in the introduction section need to be addressed

 The significance of the study has not been properly articulated, hence needs to be worked on

 Abbreviations such as MBBS, AETCOM… should be written in full first before subsequent use

 How was the following objective achieved - to provide educators with insights into students’ perspectives on body donation (See last paragraph of the introduction section) since the study was done among students only

**Method**

 The following sentence is not clear - Bachelor of Medicine, and Bachelor of Surgery from admissions of two consecutive years. Was the study done in two years? Did study use students in two levels? may year 1 and 2... Kindly clarify please to improve readership. Information about study setting is missing. Was the study done in single school? When was the study done? Duration of the study is not clear. How was the study tool designed? Who validated the questionnaire? If this was done by the authors, kindly provide mode more detail including the Cronbach alpha value. Data analysis is missing

**Result and Discussion**

 The result section is poorly presented…. The result is mostly descriptive. Struggling provide more guidance because lots of information are missing in the method section

Is the work clearly and accurately presented and does it cite the current literature?

Yes

If applicable, is the statistical analysis and its interpretation appropriate?

No

Are all the source data underlying the results available to ensure full reproducibility?

Yes

Is the study design appropriate and is the work technically sound?

Partly

Are the conclusions drawn adequately supported by the results?

Yes

Are sufficient details of methods and analysis provided to allow replication by others?

No

Reviewer Expertise:

Anatomy and Medical Education

I confirm that I have read this submission and believe that I have an appropriate level of expertise to confirm that it is of an acceptable scientific standard, however I have significant reservations, as outlined above.

# Response

**Published:** 2026-05-20

**Authors:** RAJANIGANDHA VADGAONKAR

**Affiliations:** Kasturba Medical College Mangalore, Mangaluru, Karnataka, India

Respected Sir

 Thank you immensely for reviewing our manuscript and giving valuable suggestions to improvise the content and quality. All suggestions have been taken into and incorporated in the revised version of the manuscript as per your instructions.

 The title of the manuscript has been revised as per the suggestion.

**The revised title is “Awareness and Perception of concept of body donation among the first-year medical students: A questionnaire-based study”**

  The globally acceptable terms and the highlights of the study are included.

 Abstract was revised with the addition of study location and duration.

 The inclusion criteria of the batches of the students and the conclusion part of the abstract were revised.

 The written English of the introduction section was edited.

  Significance of the study was elaborated, and the abbreviations were given in full form at the first place, when they appear.

 The objectives were revised as per the suggestion. The inclusion criteria of the batches of medical students are again clarified in the materials and methods section.

 The study setting is given in detail in this revised version of the manuscript.

 The study tool was detailed including the validation aspects like Cronbach alpha value. The data analysis section was added.

 The limitation of the study is added with respect to the results and statistical analysis.

# Reviewer report: Reviewer response for version 2

**DOI:** 10.5256/f1000research.197213.r469472

**Published:** 2026-03-23

**Authors:** Ern Ogt

**Affiliations:** 1 Istanbul Medeniyet University, Istanbul, Turkey; 2 Akdeniz University Gene and Cell Therapy Center, Antalya, Antalya, Turkey

Many points are explained several times and repetitive especially in discussion part. The discussion should be reduced and focused more directly on the main findings rather than general information. Recent studies should be added and discussed especially the importance of cadaver and body donation in anatomy education rather than models and 3d applications. It should be mentioned: early structured teaching can also improve students’ ethical awareness and knowledge of donation-related procedures: Ogut E., et al., 2025 - Ref 1. Cochran’s formula are not well explained it should be improved. p values should be written replace for descriptive statistics. Several terms are repeated unnecessarily and English needs editing. Discussion should be written from the study data.

Is the work clearly and accurately presented and does it cite the current literature?

No

If applicable, is the statistical analysis and its interpretation appropriate?

No

Are all the source data underlying the results available to ensure full reproducibility?

No

Is the study design appropriate and is the work technically sound?

No

Are the conclusions drawn adequately supported by the results?

No

Are sufficient details of methods and analysis provided to allow replication by others?

No

Reviewer Expertise:

Medical education

I confirm that I have read this submission and believe that I have an appropriate level of expertise to state that I do not consider it to be of an acceptable scientific standard, for reasons outlined above.

## References

[1] : Comprehensive evaluation of the educational impact and effectiveness of specialized study modules in cross-sectional anatomy: a study on student engagement and learning outcomes. *BMC Medical Education* .2025;25(1) : 10.1186/s12909-025-07050-9 10.1186/s12909-025-07050-9 40211255 PMC11987277

# Reviewer report: Reviewer response for version 1

**DOI:** 10.5256/f1000research.193622.r453743

**Published:** 2026-02-10

**Authors:** Rohith Motappa

**Affiliations:** 1 ESIC Medical College & PGIMSR and Model Hospital, Bangalore, Karnataka, India

General Comments

 The manuscript titled " Perception of body donation among the Phase-1 medical students, a questionnaire-based study". addresses an important and socially relevant topic, as body donation plays a crucial role in medical education. Assessing perceptions among MBBS students is important, particularly in the context of ethical awareness and cadaver-based learning. The presentation of the study require improvement to enhance clarity and impact.

 Major Comments

 1. Study Rationale - The introduction provides background on body donation, but the rationale for focusing specifically on MBBS students and not other courses in the selected institution/region should be strengthened.

 2. Methodology -The study design should be described in greater detail, including sampling technique, inclusion and exclusion criteria, and sample size justification. Institutional ethics committee approval and informed consent should be clearly mentioned.

 3. Results Presentation -Use more tables or figures. Percentages should be accompanied by absolute numbers for better clarity.

 4. Discussion -Potential reasons for positive or negative perceptions among students should be explored in more depth.

 5. Careful language editing is recommended and check on grammatical and typographical errors

 6. The conclusion could be more concise and should clearly reflect the study objectives and key findings.

 The manuscript addresses an important issue and has the potential to contribute meaningfully to medical education literature. However, few revisions are required to improve methodological clarity, depth of discussion, and overall presentation before the manuscript can be considered for indexing.

Is the work clearly and accurately presented and does it cite the current literature?

Yes

If applicable, is the statistical analysis and its interpretation appropriate?

Partly

Are all the source data underlying the results available to ensure full reproducibility?

Partly

Is the study design appropriate and is the work technically sound?

Yes

Are the conclusions drawn adequately supported by the results?

Partly

Are sufficient details of methods and analysis provided to allow replication by others?

Yes

Reviewer Expertise:

TB, NCDs

I confirm that I have read this submission and believe that I have an appropriate level of expertise to confirm that it is of an acceptable scientific standard.

# Response

**Published:** 2026-02-10

**Authors:** RAJANIGANDHA VADGAONKAR

**Affiliations:** Kasturba Medical College Mangalore, Mangaluru, Karnataka, India

Dear Reviewer

 We thank you immensely for your approval and expert comments and opinion.

 we will address your comments during the revision of the manuscript.
